# Crystal structure of 6-(*p*-tol­yl)benzo[*b*]naphtho[2,3-*d*]thio­phene and of an ortho­rhom­bic polymorph of 7-phenyl­anthra[2,3-*b*]benzo[*d*]thio­phene

**DOI:** 10.1107/S2056989016012937

**Published:** 2016-08-16

**Authors:** S. Gopinath, K. Sethusankar, Helen Stoeckli-Evans, Muhamad Rafiq, Arasambattu K. Mohanakrishnan

**Affiliations:** a Department of Physics, RKM Vivekananda College (Autonomous), Chennai 600 004, India; b University of Neuchâtel, Institute of Physics, Rue Emile-Argand 11, CH-2000 Neuchâtel, Switzerland; c Department of Organic Chemistry, University of Madras, Guindy Campus, Chennai 600 025, India

**Keywords:** crystal structure, benzo­thio­phene, benzo[*b*]naphtho­[2,3-*d*]thio­phene, anthra[2,3-*b*]benzo[*d*]thio­phene, C—H⋯π inter­actions, offset π–π inter­actions

## Abstract

The title compounds, C_23_H_16_S, (I), and C_26_H_16_S, (II), are benzo­thio­phene derivatives in which the benzo­thio­phene moiety is fused with a naphthalene ring system in (I), and with an anthracene ring system in (II). In (I), the mean plane of the benzo­thio­phene ring system makes a dihedral angle of 2.28 (6)° with the naphthalene ring system, and a dihedral angle of 1.28 (6)° with the anthracene ring system in (II), showing that the fused units are essentially planar. In (I), the 4-methyl­benzene ring substituent makes a dihedral angle of 71.40 (9)° with the naphthalene ring system, while the phenyl ring substituent in (II) makes a dihedral angle of 67.08 (12)° with the anthracene ring system. In the crystals of both compounds, mol­ecules are linked by C—H⋯π inter­actions, leading to the formation of slabs parallel to (001) in (I) and to zigzag chains along [001] in (II). There are also offset π–π inter­actions present within the slabs in (I). In the crystal of (II), they link the chains, forming sheets parallel to (010). The triclinic polymorph of compound (II) has been reported [Sivasakthikumaran *et al.*, (2012 ▸). *J. Org. Chem.* **77**, 9053–9071].

## Abstract 2

The title compounds, 6-(*p*-tol­yl)benzo[*b*]naphtho­[2,3-*d*]thio­phene and 7-phenyl­anthra[2,3-*b*]benzo[*d*]thio­phene, are benzo­thio­phene derivatives in which the benzo­thio­phene moiety is fused with a naphthalene ring system in the former and with an anthracene ring system in the latter. In the former, the 4-methyl­benzene ring substituent makes a dihedral angle of 71.40 (9)° with the mean plane of the naphthalene ring system, while the phenyl ring substituent in the latter makes a dihedral angle of 67.08 (12)° with the mean plane of the anthracene ring system.

## Chemical context   

The thio­phene nucleus has been shown to be an important heterocyclic unit in compounds possessing promising pharmacological characteristics, such as anti-HIV PR inhibitors (Bonini *et al.*, 2005 ▸) and anti-breast cancer (Brault *et al.*, 2005 ▸) activities. Benzo­thio­phenes are important biologically active mol­ecules. One of the most important drugs based on the benzo­thio­phene system is Raloxifine, used for the prevention and treatment of osteoporosis in postmenopausal women (Jordan, 2003 ▸). Benzo­thio­phenes are also present in lumin­escent components used in organic materials (Russell & Press, 1996 ▸).

Naphtho­[2,3-*b*]thio­phene derivatives have been found to exhibit anti­proliferative activity related to the inhibition of tublin polymerization (Zuse *et al.*, 2007 ▸, 2006 ▸). As a result of their outstanding electronic testability and considerable chemical and environmental stability, thio­phene derivatives have been widely used in solar cells (Justin Thomas *et al.*, 2008 ▸; Hänsel *et al.*, 2003 ▸), organic light-emitting diodes (OLEDs) (Mazzeo *et al.*, 2003 ▸), organic field-effect transistors (OFETs) (Zhan *et al.*, 2007 ▸) and as NLO devices (Bedworth *et al.*, 1996 ▸; Raposo *et al.*, 2011 ▸).Against this background, we describe herein the syntheses and crystal structures of the title benzo­thio­phene derivatives. 
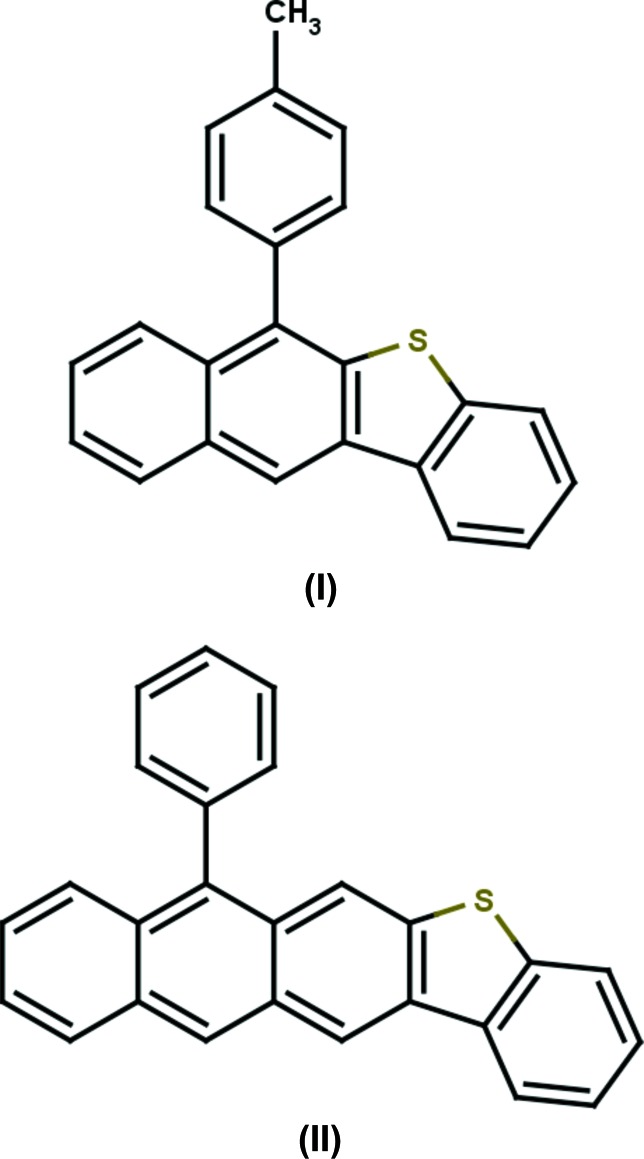


## Structural commentary   

**Figure 1:**
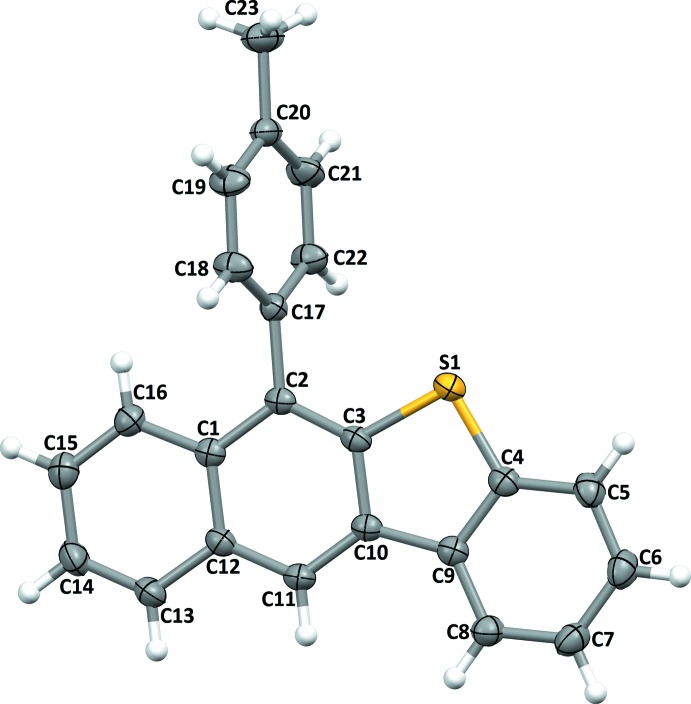
The mol­ecular structure of compound (I), showing the atom labelling. Displacement ellipsoids are drawn at the 30% probability level.

The mol­ecular structures of the title compounds, (I) and (II), are illustrated in Figs. 1 ▸ and 2 ▸, respectively. In both compounds, the benzo­thio­phene ring systems are almost planar with the dihedral angles between the benzene and thio­phene rings being 1.85 (11)° in (I) and 0.56 (18)° in (II).

In compound (I), the naphthalene ring system (atoms C1–C3/C10–C16) (r.m.s. deviation = 0.006 Å) makes a a dihedral angle of 2.28 (6)° with the benzo­thio­phene (C3–C10/S1) ring system (r.m.s. deviation = 0.023 Å). The 4-methyl­benzene ring substituent (C17–C22) makes a dihedral angle of 71.40 (9)° with the naphthalene ring system

In compound (II), the anthracene ring system (C1–C3/C10–C20) is almost planar (r.m.s. deviation = 0.075 Å) and makes a a dihedral angle of 7.31 (9)° with the benzo­thio­phene (C3–C10/S1) ring system (r.m.s. deviation = 0.012 Å). Here, the phenyl ring substituent (C21–C26) in (II) makes a dihedral angle of 67.08 (12)° with the anthracene ring system, and the anthracene ring is (−)anti­periplanar with respect to the benzo­thio­phene moiety, as indicated by the S1—C3—C10—C11 torsion angle of −176.4 (2)°.

In the triclinic polymorph of compound (II) (Sivasakthikumaran *et al.*, 2012 ▸), the major component of the disordered phenyl ring substituent makes a dihedral angle of 79.39 (12)° with the anthracene ring system.

## Supra­molecular features   

**Figure 3:**
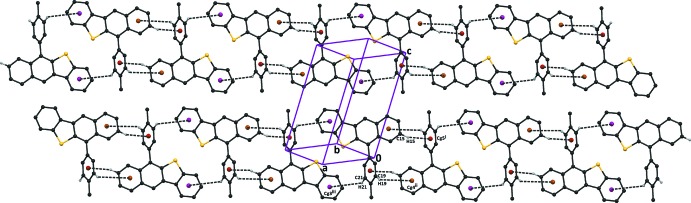
The crystal packing of compound (I). The C—H⋯π inter­actions are shown as dashed lines (see Table 1 ▸ for details). H atoms not involved in these inter­actions have been omitted for clarity.

In the crystals of both compounds, mol­ecules are linked by C—H⋯π inter­actions (see Tables 1 ▸ and 2 ▸), leading to the formation of slabs parallel to (001) in (I), and to zigzag chains along [001] in (II); as illustrated in Figs. 3 ▸, 4 ▸ and 5 ▸. There are also offset π–π inter­actions present within the slabs in (I) [*Cg*1⋯*Cg*3^i^ = 3.629 (1) Å, inter­planar distance = 3.602 (1) Å, slippage = 0.49 Å; *Cg*2⋯*Cg*4^ii^ = 3.983 (1), inter­planar distance = 3.473 (1), slippage 1.79 Å; *Cg*1, *Cg*2, *Cg*3 and *Cg*4 are the centroids of rings S1/C3/C4/C9/C10, C1–C3/C10–C12, C1/C12–C16 and C4–C9, respectively; symmetry codes: (i) *x* + 1, *y*, *z*; (ii) *x* − 1, *y*, *z*]. In the crystal of (II), offset π–π inter­actions link the chains, forming sheets parallel to (010) [*Cg*2⋯*Cg*4^iii^ = 3.711 (2) Å, inter­planar distance = 3.479 (1) Å, slippage = 1.21 Å; *Cg*3⋯*Cg*4^iii^ = 3.741 (2) Å, inter­planar distance = 3.443 (1) Å, slippage = 1.22 Å; *Cg*2, *Cg*3 and *Cg*4 are the centroids of rings C1–C3/C10–C12, C1/C12–C16 and C4–C9, respectively; symmetry code: (iii) −*x* + 1, −*y* + 1, −*z* + 1].

## Database survey   

A search of the Cambridge Structural Database (Version 5.38, update May 2016; Groom *et al.*, 2016 ▸) for the naphtho­benzo­thio­phene skeleton gave 32 hits. Among these there are five naphtho­benzo­thio­phene derivatives that resemble compound (I), *viz.* 6-(phen­yl)benzo[*b*]naphtho­[2,3-*d*]thio­phene (NEQMAZ; Silambarasan *et al.*, 2013 ▸), 6-(4-meth­oxy­phen­yl)benzo[*b*]naphtho­[2,3-*d*]thio­phene (PECQEV; Silambarasan *et al.*, 2012 ▸), 6-(2-thien­yl)benzo[*b*]naphtho­[2,3-*d*]thiophene (XIMZUQ; Sivasakthikumaran *et al.*, 2012 ▸), 6-(1-benzo­thio­phen-3-yl)benzo[*b*]naphtho­[2,3-*d*]thio­phene (HIXQUB; Li *et al.*, 2007 ▸) and 1,3-di­methyl­benzo[*b*]naphtho­[2,3-*d*]thio­phene (ROMPUF/ROMPUF01; Umarani *et al.*, 2009/Dhayalan *et al.*, 2009 ▸). There are also two anthracene analogues, *viz.* anthra[2,3-*b*]benzo[*d*]thio­phene itself (JOHSOP; Du *et al.*, 2008 ▸), and 7-(1-benzo­thio­phen-2-yl)anthra[2,3-*b*]benzo[*d*]thio­phene (FOLGEU; Rafiq *et al.*, 2014 ▸); as well as the triclinic polymorph of compound (II) (XIMZOK; Sivasakthikumaran *et al.*, 2012 ▸).

## Synthesis and crystallization   

**Compound (I)**

The reduction of the diketone (benzo­thio­phen-3-yl)[2-(4-methyl­benzo­yl)phen­yl]methanone (0.85 g, 2.38 mmol) using sodium borohydride (0.49 g, 12.89 mmol) followed by work-up gave the diol. Dipivaloylation of the diol (0.77 g, 2.31 mmol) using pivaloyl chloride (1.39 g, 11.52 mmol) and tri­ethyl­amine (4.69 g, 45.20 mmol) in the presence of a catalytic amount of DMAP (10 mg) in dry DCM (20 ml) led to the isolation of dipivalate (benzo[*b*]thio­phen-3-yl){2-[pivalo­yloxy(*p*-tol­yl) meth­yl]phen­yl}methyl pivalate as a viscous liquid. Dipivalate (benzo[*b*]thio­phen-3-yl){2-[pivalo­yloxy(*p*-to­yl) meth­yl]phen­yl}methyl pivalate (0.98 g, 1.96 mmol) upon inter­action with ZnBr_2_ (0.02 g, 0.13 mmol) followed by removal of solvent and column chromatographic purification (silica gel; hexa­ne–ethyl acetate, 99:1) gave 6-(*p*-tol­yl)benzo[*b*]naphtho­[2,3-*d*]thiophene as a pale-green solid (yield 0.53 g, 78%). Single crystals suitable for X-ray diffraction were prepared by slow evaporation of a solution of (I) in ethyl acetate at room temperature (m.p. 391–393 K).

**Compound (II)**

The reduction of the diketone (2-benzoyl­phen­yl)(dibenzo[*b*,*d*]thio­phen-2-yl)methanone (1.11 g, 2.38 mmol) using sodium borohydride (0.53 g, 13.94 mmol) followed by work-up gave the diol. Dipivaloylation of the diol (1.12 g, 2.82 mmol) using pivaloyl chloride (1.70 g, 14.14 mmol) and tri­ethyl­amine (5.72 g, 56.56 mmol) in the presence of a catalytic amount of DMAP (10 mg) in dry DCM (20 ml) led to the isolation of dipivalate (dibenzo[*b*,*d*]thio­phen-2-yl){2-[phen­yl(pivalo­yloxy)meth­yl]phen­yl}methyl pivalate as a thick liquid. Dipivalate (dibenzo[*b*,*d*]thio­phen-2-yl){2-[phen­yl(pivalo­yloxy)meth­yl]phen­yl}methyl pivalate (1.28 g, 2.26 mmol) upon inter­action with ZnBr_2_ (0.02 g, 0.13 mmol) followed by removal of solvent and column chromatographic purification (silica gel; hexa­ne–ethyl acetate, 99:1) gave a new ortho­rhom­bic polymorph of 7-phenyl­anthra[2,3-*b*]benzo[*d*]thio­phene (yield 0.83 g, 72%) as a yellow solid. Single crystals suitable for X-ray diffraction were prepared by slow evaporation of a solution of the compound (II) in ethyl acetate at room temperature (m.p. 463–465 K).

## Refinement   

Crystal data, data collection and structure refinement details for compounds (I) and (II) are summarized in Table 3 ▸. The C-bound H atoms were included in calculated positions and treated as riding atoms, with C—H = 0.93–0.96 Å and with *U*_iso_(H) = 1.5*U*_eq_(methyl C) and 1.2*U*_eq_(C) for other H atoms.

## Supplementary Material

Crystal structure: contains datablock(s) I, II, global. DOI: 10.1107/S2056989016012937/lh5819sup1.cif

Structure factors: contains datablock(s) I. DOI: 10.1107/S2056989016012937/lh5819Isup2.hkl

Structure factors: contains datablock(s) II. DOI: 10.1107/S2056989016012937/lh5819IIsup3.hkl

Supporting information file. DOI: 10.1107/S2056989016012937/lh5819Isup4.cml

CCDC references: 1498519, 1498518

Additional supporting information:  crystallographic information; 3D view; checkCIF report

## Figures and Tables

**Figure 2:**
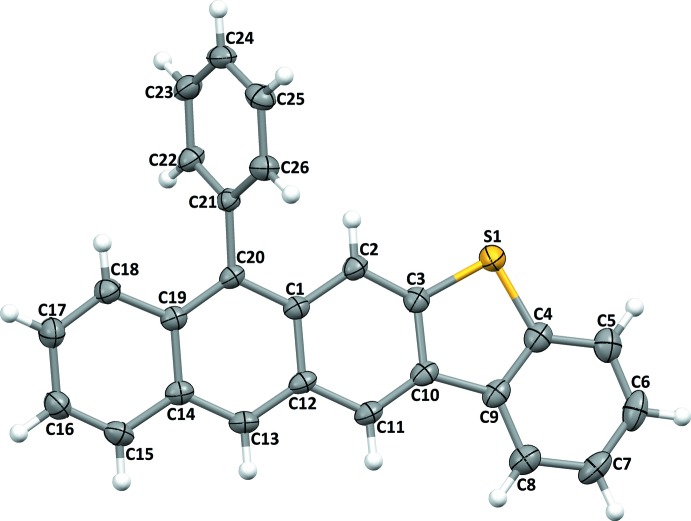
The mol­ecular structure of compound (II), showing the atom labelling. Displacement ellipsoids are drawn at the 30% probability level.

**Figure 4:**
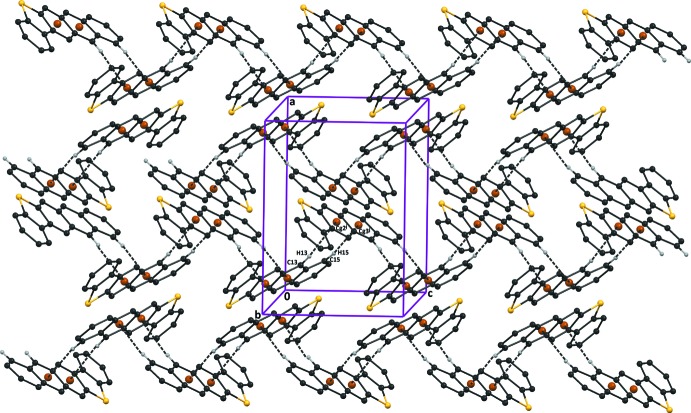
The crystal packing of compound (II), viewed along the *b* axis. The C—H⋯π inter­actions are shown as dashed lines (see Table 2 ▸ for details) and the centroids as brown balls. H atoms not involved in these inter­actions have been omitted for clarity.

**Figure 5:**
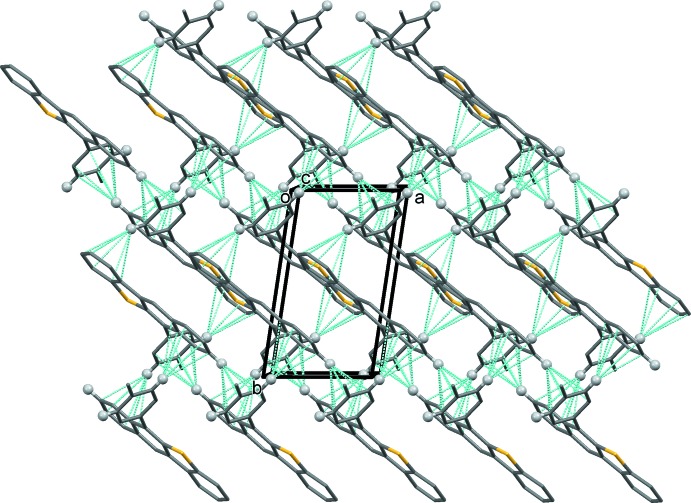
The crystal packing of compound (I), viewed along the *c* axis, showing the C—H⋯π inter­actions (represented as turquoise lines) leading to the formation of slabs parallel to (001).

**Table 1:** Hydrogen-bond geometry (Å, °) for (I) *Cg*3, *Cg*4 and *Cg*5 are the centroids of rings (C1/C12–C16), (C4–C6) and (C17–C22), respectively.

*D*—H⋯*A*	*D*—H	H⋯*A*	*D*⋯*A*	*D*—H⋯*A*
C15—H15⋯*Cg*5^i^	0.93	2.94	3.763 (3)	148
C19—H19⋯*Cg*4^ii^	0.93	2.94	3.753 (3)	147
C21—H21⋯*Cg*3^iii^	0.93	2.91	3.721 (3)	146

Symmetry codes: (i) 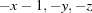; (ii) 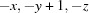; (iii) 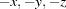.

**Table 2:** Hydrogen-bond geometry (Å, °) for (II) *Cg*2 and *Cg*3 are the centroids of rings (C1–C3/C10–C12) and (C1/C12–C14/C19/C20), respectively.

*D*—H⋯*A*	*D*—H	H⋯*A*	*D*⋯*A*	*D*—H⋯*A*
C13—H13⋯*Cg*2^i^	0.93	2.97	3.885 (4)	168
C15—H15⋯*Cg*3^i^	0.93	2.57	3.479 (4)	166

Symmetry code: (i) 
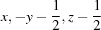
.

**Table 3:** Experimental details

	(I)	(II)
Crystal data		
Chemical formula	C_23_H_16_S	C_26_H_16_S
*M*_r_	324.42	360.45
Crystal system, space group	Triclinic, *P* 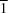	Orthorhombic, *P* *c* *c* *n*
Temperature (K)	296	296
*a*, *b*, *c* (Å)	6.2404 (3), 11.1725 (6), 12.9987 (7)	12.2159 (8), 33.1138 (4), 8.8993 (5)
α, β, γ (°)	109.284 (2), 100.233 (4), 93.925 (2)	90, 90, 90
*V* (Å^3^)	833.90 (8)	3599.9 (3)
*Z*	2	8
Radiation type	Mo *K*α	Mo *K*α
μ (mm^−1^)	0.19	0.19
Crystal size (mm)	0.30 × 0.25 × 0.20	0.30 × 0.25 × 0.25

Data collection		
Diffractometer	Bruker Kappa APEXII CCD	Bruker Kappa APEXII CCD
Absorption correction	Multi-scan (*SADABS*; Bruker, 2008 ▸)	Multi-scan (*SADABS*; Bruker, 2008 ▸)
*T*_min_, *T*_max_	0.944, 0.962	0.946, 0.955
No. of measured, independent and observed [*I* > 2σ(*I*)] reflections	15861, 2944, 2407	43542, 3171, 2540
*R*_int_	0.024	0.036
(sin θ/λ)_max_ (Å^−1^)	0.595	0.595

Refinement		
*R*[*F* ^2^ > 2σ(*F* ^2^)], *wR*(*F* ^2^), *S*	0.039, 0.113, 1.07	0.059, 0.182, 1.04
No. of reflections	2944	3171
No. of parameters	218	244
H-atom treatment	H-atom parameters constrained	H-atom parameters constrained
Δρ_max_, Δρ_min_ (e Å^−3^)	0.21, −0.21	1.06, −0.40

Computer programs: *APEX2* and *SAINT* (Bruker, 2008 ▸), *SHELXS97* and *SHELXL97* (Sheldrick, 2008 ▸), *ORTEP-3 for Windows* (Farrugia, 2012 ▸), *Mercury* (Macrae *et al.*, 2008 ▸) and *PLATON* (Spek, 2009 ▸).

## supplementary crystallographic information

### (I) 6-(p-Tolyl)benzo[b]naphtho[2,3-d]thiophene Crystal data

**Table:**

C_23_H_16_S	*Z* = 2
*M**_r_* = 324.42	*F*(000) = 340
Triclinic, *P*1	*D*_x_ = 1.292 Mg m^−^^3^
Hall symbol: -P 1	Mo *K*α radiation, λ = 0.71073 Å
*a* = 6.2404 (3) Å	Cell parameters from 2944 reflections
*b* = 11.1725 (6) Å	θ = 2.1–25.0°
*c* = 12.9987 (7) Å	µ = 0.19 mm^−^^1^
α = 109.284 (2)°	*T* = 296 K
β = 100.233 (4)°	Block, colourless
γ = 93.925 (2)°	0.30 × 0.25 × 0.20 mm
*V* = 833.90 (8) Å^3^	

### (I) 6-(p-Tolyl)benzo[b]naphtho[2,3-d]thiophene Data collection

**Table:**

Bruker Kappa APEXII CCD diffractometer	2944 independent reflections
Radiation source: fine-focus sealed tube	2407 reflections with *I* > 2σ(*I*)
Graphite monochromator	*R*_int_ = 0.024
ω & φ scans	θ_max_ = 25.0°, θ_min_ = 2.1°
Absorption correction: multi-scan (SADABS; Bruker, 2008)	*h* = −7→7
*T*_min_ = 0.944, *T*_max_ = 0.962	*k* = −13→13
15861 measured reflections	*l* = −15→15

### (I) 6-(p-Tolyl)benzo[b]naphtho[2,3-d]thiophene Refinement

**Table:**

Refinement on *F*^2^	Primary atom site location: structure-invariant direct methods
Least-squares matrix: full	Secondary atom site location: difference Fourier map
*R*[*F*^2^ > 2σ(*F*^2^)] = 0.039	Hydrogen site location: inferred from neighbouring sites
*wR*(*F*^2^) = 0.113	H-atom parameters constrained
*S* = 1.07	*w* = 1/[σ^2^(*F*_o_^2^) + (0.0484*P*)^2^ + 0.4189*P*] where *P* = (*F*_o_^2^ + 2*F*_c_^2^)/3
2944 reflections	(Δ/σ)_max_ = 0.002
218 parameters	Δρ_max_ = 0.21 e Å^−^^3^
0 restraints	Δρ_min_ = −0.21 e Å^−^^3^

### (I) 6-(p-Tolyl)benzo[b]naphtho[2,3-d]thiophene Special details

**Table:**

Geometry. All esds (except the esd in the dihedral angle between two l.s. planes) are estimated using the full covariance matrix. The cell esds are taken into account individually in the estimation of esds in distances, angles and torsion angles; correlations between esds in cell parameters are only used when they are defined by crystal symmetry. An approximate (isotropic) treatment of cell esds is used for estimating esds involving l.s. planes.
Refinement. Refinement of F^2^ against ALL reflections. The weighted R-factor wR and goodness of fit S are based on F^2^, conventional R-factors R are based on F, with F set to zero for negative F^2^. The threshold expression of F^2^ > 2sigma(F^2^) is used only for calculating R-factors(gt) etc. and is not relevant to the choice of reflections for refinement. R-factors based on F^2^ are statistically about twice as large as those based on F, and R- factors based on ALL data will be even larger.

### (I) 6-(p-Tolyl)benzo[b]naphtho[2,3-d]thiophene Fractional atomic coordinates and isotropic or equivalent isotropic displacement parameters (Å^2^)

**Table:**

	*x*	*y*	*z*	*U*_iso_*/*U*_eq_	
C1	−0.1614 (3)	0.24648 (18)	0.15929 (16)	0.0358 (4)	
C2	−0.0247 (3)	0.27515 (17)	0.09112 (15)	0.0344 (4)	
C3	0.1606 (3)	0.36306 (18)	0.14305 (15)	0.0355 (4)	
C4	0.5130 (3)	0.51269 (19)	0.20179 (16)	0.0401 (5)	
C5	0.7116 (3)	0.5852 (2)	0.21532 (19)	0.0504 (5)	
H5	0.7718	0.5822	0.1541	0.060*	
C6	0.8178 (4)	0.6618 (2)	0.3211 (2)	0.0566 (6)	
H6	0.9518	0.7108	0.3316	0.068*	
C7	0.7283 (4)	0.6671 (2)	0.4127 (2)	0.0551 (6)	
H7	0.8012	0.7209	0.4835	0.066*	
C8	0.5328 (4)	0.5937 (2)	0.39951 (18)	0.0474 (5)	
H8	0.4743	0.5970	0.4612	0.057*	
C9	0.4228 (3)	0.51430 (18)	0.29316 (16)	0.0380 (4)	
C10	0.2200 (3)	0.42696 (18)	0.26060 (15)	0.0360 (4)	
C11	0.0889 (3)	0.39891 (19)	0.32582 (16)	0.0403 (5)	
H11	0.1264	0.4399	0.4028	0.048*	
C12	−0.1012 (3)	0.30925 (19)	0.27797 (16)	0.0390 (4)	
C13	−0.2388 (4)	0.2784 (2)	0.34417 (18)	0.0485 (5)	
H13	−0.2009	0.3178	0.4213	0.058*	
C14	−0.4235 (4)	0.1932 (2)	0.2978 (2)	0.0544 (6)	
H14	−0.5116	0.1752	0.3430	0.065*	
C15	−0.4829 (4)	0.1316 (2)	0.1815 (2)	0.0513 (5)	
H15	−0.6102	0.0729	0.1500	0.062*	
C16	−0.3550 (3)	0.1575 (2)	0.11515 (18)	0.0437 (5)	
H16	−0.3963	0.1154	0.0384	0.052*	
C17	−0.0765 (3)	0.21177 (18)	−0.03270 (15)	0.0359 (4)	
C18	−0.2468 (4)	0.2399 (2)	−0.10000 (18)	0.0514 (6)	
H18	−0.3361	0.2983	−0.0676	0.062*	
C19	−0.2881 (4)	0.1835 (2)	−0.21439 (18)	0.0560 (6)	
H19	−0.4042	0.2049	−0.2576	0.067*	
C20	−0.1618 (4)	0.0964 (2)	−0.26596 (17)	0.0467 (5)	
C21	0.0061 (4)	0.0673 (2)	−0.19921 (19)	0.0565 (6)	
H21	0.0935	0.0079	−0.2319	0.068*	
C22	0.0493 (4)	0.1237 (2)	−0.08442 (18)	0.0514 (6)	
H22	0.1652	0.1019	−0.0415	0.062*	
C23	−0.2067 (5)	0.0357 (3)	−0.3913 (2)	0.0739 (8)	
H23A	−0.0779	0.0023	−0.4136	0.111*	
H23B	−0.2443	0.0987	−0.4243	0.111*	
H23C	−0.3267	−0.0327	−0.4158	0.111*	
S1	0.35084 (8)	0.41019 (5)	0.07515 (4)	0.04430 (18)	

### (I) 6-(p-Tolyl)benzo[b]naphtho[2,3-d]thiophene Atomic displacement parameters (Å^2^)

**Table:**

	*U*^11^	*U*^22^	*U*^33^	*U*^12^	*U*^13^	*U*^23^
C1	0.0382 (10)	0.0343 (10)	0.0370 (10)	0.0107 (8)	0.0085 (8)	0.0139 (8)
C2	0.0387 (10)	0.0344 (10)	0.0318 (10)	0.0126 (8)	0.0081 (8)	0.0119 (8)
C3	0.0390 (10)	0.0391 (11)	0.0328 (10)	0.0127 (8)	0.0120 (8)	0.0146 (8)
C4	0.0435 (11)	0.0410 (11)	0.0404 (11)	0.0097 (9)	0.0095 (9)	0.0192 (9)
C5	0.0487 (12)	0.0581 (14)	0.0534 (13)	0.0031 (10)	0.0127 (10)	0.0311 (11)
C6	0.0513 (13)	0.0559 (14)	0.0643 (16)	−0.0075 (11)	0.0046 (11)	0.0300 (12)
C7	0.0601 (14)	0.0463 (13)	0.0496 (13)	−0.0078 (11)	−0.0010 (11)	0.0143 (11)
C8	0.0559 (13)	0.0450 (12)	0.0399 (11)	0.0026 (10)	0.0093 (10)	0.0142 (9)
C9	0.0420 (10)	0.0360 (10)	0.0385 (11)	0.0083 (8)	0.0094 (8)	0.0153 (9)
C10	0.0407 (10)	0.0347 (10)	0.0338 (10)	0.0101 (8)	0.0086 (8)	0.0120 (8)
C11	0.0456 (11)	0.0430 (11)	0.0311 (10)	0.0065 (9)	0.0098 (8)	0.0103 (9)
C12	0.0417 (10)	0.0397 (11)	0.0388 (11)	0.0102 (9)	0.0131 (9)	0.0147 (9)
C13	0.0532 (12)	0.0554 (13)	0.0408 (12)	0.0066 (10)	0.0190 (10)	0.0172 (10)
C14	0.0510 (13)	0.0619 (15)	0.0585 (14)	0.0030 (11)	0.0239 (11)	0.0263 (12)
C15	0.0446 (12)	0.0515 (13)	0.0598 (14)	0.0018 (10)	0.0106 (10)	0.0233 (11)
C16	0.0433 (11)	0.0431 (12)	0.0429 (11)	0.0047 (9)	0.0054 (9)	0.0149 (9)
C17	0.0370 (10)	0.0360 (10)	0.0350 (10)	0.0056 (8)	0.0092 (8)	0.0118 (8)
C18	0.0581 (13)	0.0569 (14)	0.0416 (12)	0.0271 (11)	0.0119 (10)	0.0157 (10)
C19	0.0624 (14)	0.0656 (15)	0.0406 (12)	0.0183 (12)	0.0021 (11)	0.0218 (11)
C20	0.0572 (13)	0.0432 (12)	0.0350 (11)	−0.0068 (10)	0.0102 (10)	0.0100 (9)
C21	0.0612 (14)	0.0553 (14)	0.0470 (13)	0.0184 (11)	0.0196 (11)	0.0039 (11)
C22	0.0496 (12)	0.0575 (14)	0.0426 (12)	0.0214 (11)	0.0069 (10)	0.0103 (10)
C23	0.100 (2)	0.0677 (17)	0.0405 (13)	−0.0129 (15)	0.0131 (13)	0.0077 (12)
S1	0.0469 (3)	0.0525 (3)	0.0360 (3)	0.0051 (2)	0.0137 (2)	0.0165 (2)

### (I) 6-(p-Tolyl)benzo[b]naphtho[2,3-d]thiophene Geometric parameters (Å, º)

**Table:**

C1—C16	1.411 (3)	C12—C13	1.420 (3)
C1—C2	1.426 (3)	C13—C14	1.349 (3)
C1—C12	1.434 (3)	C13—H13	0.9300
C2—C3	1.373 (3)	C14—C15	1.405 (3)
C2—C17	1.492 (3)	C14—H14	0.9300
C3—C10	1.423 (3)	C15—C16	1.358 (3)
C3—S1	1.7492 (19)	C15—H15	0.9300
C4—C5	1.384 (3)	C16—H16	0.9300
C4—C9	1.397 (3)	C17—C18	1.375 (3)
C4—S1	1.746 (2)	C17—C22	1.376 (3)
C5—C6	1.372 (3)	C18—C19	1.377 (3)
C5—H5	0.9300	C18—H18	0.9300
C6—C7	1.388 (3)	C19—C20	1.370 (3)
C6—H6	0.9300	C19—H19	0.9300
C7—C8	1.373 (3)	C20—C21	1.366 (3)
C7—H7	0.9300	C20—C23	1.508 (3)
C8—C9	1.393 (3)	C21—C22	1.382 (3)
C8—H8	0.9300	C21—H21	0.9300
C9—C10	1.449 (3)	C22—H22	0.9300
C10—C11	1.369 (3)	C23—H23A	0.9600
C11—C12	1.400 (3)	C23—H23B	0.9600
C11—H11	0.9300	C23—H23C	0.9600
			
C16—C1—C2	122.80 (18)	C14—C13—H13	119.2
C16—C1—C12	117.81 (17)	C12—C13—H13	119.2
C2—C1—C12	119.39 (17)	C13—C14—C15	120.2 (2)
C3—C2—C1	117.87 (17)	C13—C14—H14	119.9
C3—C2—C17	120.13 (17)	C15—C14—H14	119.9
C1—C2—C17	121.99 (17)	C16—C15—C14	120.2 (2)
C2—C3—C10	123.09 (17)	C16—C15—H15	119.9
C2—C3—S1	125.07 (15)	C14—C15—H15	119.9
C10—C3—S1	111.84 (14)	C15—C16—C1	121.9 (2)
C5—C4—C9	121.40 (19)	C15—C16—H16	119.1
C5—C4—S1	125.91 (16)	C1—C16—H16	119.1
C9—C4—S1	112.68 (15)	C18—C17—C22	117.15 (19)
C6—C5—C4	118.5 (2)	C18—C17—C2	122.18 (17)
C6—C5—H5	120.8	C22—C17—C2	120.65 (17)
C4—C5—H5	120.8	C17—C18—C19	121.4 (2)
C5—C6—C7	121.0 (2)	C17—C18—H18	119.3
C5—C6—H6	119.5	C19—C18—H18	119.3
C7—C6—H6	119.5	C20—C19—C18	121.4 (2)
C8—C7—C6	120.6 (2)	C20—C19—H19	119.3
C8—C7—H7	119.7	C18—C19—H19	119.3
C6—C7—H7	119.7	C21—C20—C19	117.3 (2)
C7—C8—C9	119.6 (2)	C21—C20—C23	121.5 (2)
C7—C8—H8	120.2	C19—C20—C23	121.2 (2)
C9—C8—H8	120.2	C20—C21—C22	121.7 (2)
C8—C9—C4	118.95 (19)	C20—C21—H21	119.1
C8—C9—C10	128.82 (18)	C22—C21—H21	119.1
C4—C9—C10	112.23 (17)	C17—C22—C21	121.0 (2)
C11—C10—C3	118.90 (18)	C17—C22—H22	119.5
C11—C10—C9	129.40 (18)	C21—C22—H22	119.5
C3—C10—C9	111.69 (17)	C20—C23—H23A	109.5
C10—C11—C12	120.75 (18)	C20—C23—H23B	109.5
C10—C11—H11	119.6	H23A—C23—H23B	109.5
C12—C11—H11	119.6	C20—C23—H23C	109.5
C11—C12—C13	121.63 (19)	H23A—C23—H23C	109.5
C11—C12—C1	120.00 (17)	H23B—C23—H23C	109.5
C13—C12—C1	118.37 (18)	C4—S1—C3	91.52 (9)
C14—C13—C12	121.6 (2)		
			
C16—C1—C2—C3	−179.76 (17)	C10—C11—C12—C1	−0.5 (3)
C12—C1—C2—C3	−0.2 (3)	C16—C1—C12—C11	−179.63 (17)
C16—C1—C2—C17	−0.5 (3)	C2—C1—C12—C11	0.8 (3)
C12—C1—C2—C17	179.12 (16)	C16—C1—C12—C13	0.1 (3)
C1—C2—C3—C10	−0.6 (3)	C2—C1—C12—C13	−179.48 (18)
C17—C2—C3—C10	−179.96 (16)	C11—C12—C13—C14	179.1 (2)
C1—C2—C3—S1	179.27 (13)	C1—C12—C13—C14	−0.6 (3)
C17—C2—C3—S1	−0.1 (3)	C12—C13—C14—C15	0.6 (4)
C9—C4—C5—C6	−1.3 (3)	C13—C14—C15—C16	−0.1 (3)
S1—C4—C5—C6	179.61 (17)	C14—C15—C16—C1	−0.4 (3)
C4—C5—C6—C7	−0.3 (3)	C2—C1—C16—C15	179.98 (18)
C5—C6—C7—C8	1.3 (4)	C12—C1—C16—C15	0.4 (3)
C6—C7—C8—C9	−0.7 (3)	C3—C2—C17—C18	−108.5 (2)
C7—C8—C9—C4	−0.9 (3)	C1—C2—C17—C18	72.2 (3)
C7—C8—C9—C10	178.1 (2)	C3—C2—C17—C22	70.1 (3)
C5—C4—C9—C8	1.9 (3)	C1—C2—C17—C22	−109.1 (2)
S1—C4—C9—C8	−178.87 (15)	C22—C17—C18—C19	−0.7 (3)
C5—C4—C9—C10	−177.24 (18)	C2—C17—C18—C19	177.9 (2)
S1—C4—C9—C10	2.0 (2)	C17—C18—C19—C20	0.3 (4)
C2—C3—C10—C11	0.9 (3)	C18—C19—C20—C21	0.4 (4)
S1—C3—C10—C11	−179.04 (15)	C18—C19—C20—C23	−179.5 (2)
C2—C3—C10—C9	179.87 (17)	C19—C20—C21—C22	−0.6 (4)
S1—C3—C10—C9	0.0 (2)	C23—C20—C21—C22	179.3 (2)
C8—C9—C10—C11	−1.4 (3)	C18—C17—C22—C21	0.5 (3)
C4—C9—C10—C11	177.64 (19)	C2—C17—C22—C21	−178.2 (2)
C8—C9—C10—C3	179.71 (19)	C20—C21—C22—C17	0.2 (4)
C4—C9—C10—C3	−1.2 (2)	C5—C4—S1—C3	177.47 (19)
C3—C10—C11—C12	−0.3 (3)	C9—C4—S1—C3	−1.69 (15)
C9—C10—C11—C12	−179.05 (18)	C2—C3—S1—C4	−178.95 (17)
C10—C11—C12—C13	179.71 (18)	C10—C3—S1—C4	0.96 (14)

### (I) 6-(p-Tolyl)benzo[b]naphtho[2,3-d]thiophene Hydrogen-bond geometry (Å, º)

Cg3, Cg4 and Cg5 are the centroids of rings (C1/C12–C16), (C4–C6) and (C17–C22), respectively.

**Table:**

*D*—H···*A*	*D*—H	H···*A*	*D*···*A*	*D*—H···*A*
C15—H15···*Cg*5^i^	0.93	2.94	3.763 (3)	148
C19—H19···*Cg*4^ii^	0.93	2.94	3.753 (3)	147
C21—H21···*Cg*3^iii^	0.93	2.91	3.721 (3)	146

Symmetry codes: (i) −*x*−1, −*y*, −*z*; (ii) −*x*, −*y*+1, −*z*; (iii) −*x*, −*y*, −*z*.

### (II) 7-Phenylanthra[2,3-b]benzo[d]thiophene Crystal data

**Table:**

C_26_H_16_S	*F*(000) = 1504
*M**_r_* = 360.45	*D*_x_ = 1.330 Mg m^−^^3^
Orthorhombic, *P**c**c**n*	Mo *K*α radiation, λ = 0.71073 Å
Hall symbol: -P 2ab 2ac	Cell parameters from 3171 reflections
*a* = 12.2159 (8) Å	θ = 2.5–25.0°
*b* = 33.1138 (4) Å	µ = 0.19 mm^−^^1^
*c* = 8.8993 (5) Å	*T* = 296 K
*V* = 3599.9 (3) Å^3^	Block, colourless
*Z* = 8	0.30 × 0.25 × 0.25 mm

### (II) 7-Phenylanthra[2,3-b]benzo[d]thiophene Data collection

**Table:**

Bruker Kappa APEXII CCD diffractometer	3171 independent reflections
Radiation source: fine-focus sealed tube	2540 reflections with *I* > 2σ(*I*)
Graphite monochromator	*R*_int_ = 0.036
ω & φ scans	θ_max_ = 25.0°, θ_min_ = 2.5°
Absorption correction: multi-scan (SADABS; Bruker, 2008)	*h* = −14→14
*T*_min_ = 0.946, *T*_max_ = 0.955	*k* = −39→39
43542 measured reflections	*l* = −10→9

### (II) 7-Phenylanthra[2,3-b]benzo[d]thiophene Refinement

**Table:**

Refinement on *F*^2^	Primary atom site location: structure-invariant direct methods
Least-squares matrix: full	Secondary atom site location: difference Fourier map
*R*[*F*^2^ > 2σ(*F*^2^)] = 0.059	Hydrogen site location: inferred from neighbouring sites
*wR*(*F*^2^) = 0.182	H-atom parameters constrained
*S* = 1.04	*w* = 1/[σ^2^(*F*_o_^2^) + (0.0831*P*)^2^ + 5.3659*P*] where *P* = (*F*_o_^2^ + 2*F*_c_^2^)/3
3171 reflections	(Δ/σ)_max_ < 0.001
244 parameters	Δρ_max_ = 1.06 e Å^−^^3^
0 restraints	Δρ_min_ = −0.40 e Å^−^^3^

### (II) 7-Phenylanthra[2,3-b]benzo[d]thiophene Special details

**Table:**

Geometry. All esds (except the esd in the dihedral angle between two l.s. planes) are estimated using the full covariance matrix. The cell esds are taken into account individually in the estimation of esds in distances, angles and torsion angles; correlations between esds in cell parameters are only used when they are defined by crystal symmetry. An approximate (isotropic) treatment of cell esds is used for estimating esds involving l.s. planes.
Refinement. Refinement of F^2^ against ALL reflections. The weighted R-factor wR and goodness of fit S are based on F^2^, conventional R-factors R are based on F, with F set to zero for negative F^2^. The threshold expression of F^2^ > 2sigma(F^2^) is used only for calculating R-factors(gt) etc. and is not relevant to the choice of reflections for refinement. R-factors based on F^2^ are statistically about twice as large as those based on F, and R- factors based on ALL data will be even larger.

### (II) 7-Phenylanthra[2,3-b]benzo[d]thiophene Fractional atomic coordinates and isotropic or equivalent isotropic displacement parameters (Å^2^)

**Table:**

	*x*	*y*	*z*	*U*_iso_*/*U*_eq_	
C1	0.4387 (2)	0.40078 (9)	0.4515 (3)	0.0366 (7)	
C2	0.4905 (3)	0.42302 (9)	0.3361 (3)	0.0412 (7)	
H2	0.5423	0.4107	0.2749	0.049*	
C3	0.4643 (3)	0.46257 (9)	0.3144 (3)	0.0422 (7)	
C4	0.4365 (3)	0.53427 (10)	0.2336 (4)	0.0494 (8)	
C5	0.4359 (3)	0.57228 (11)	0.1701 (4)	0.0581 (9)	
H5	0.4836	0.5788	0.0923	0.070*	
C6	0.3634 (4)	0.60010 (11)	0.2244 (5)	0.0667 (11)	
H6	0.3614	0.6257	0.1819	0.080*	
C7	0.2930 (3)	0.59091 (11)	0.3413 (5)	0.0626 (11)	
H7	0.2442	0.6103	0.3762	0.075*	
C8	0.2948 (3)	0.55284 (10)	0.4065 (4)	0.0532 (9)	
H8	0.2479	0.5466	0.4855	0.064*	
C9	0.3679 (3)	0.52414 (9)	0.3515 (4)	0.0454 (8)	
C10	0.3829 (3)	0.48249 (9)	0.4034 (4)	0.0416 (7)	
C11	0.3348 (3)	0.46241 (9)	0.5176 (4)	0.0442 (8)	
H11	0.2827	0.4754	0.5764	0.053*	
C12	0.3627 (2)	0.42151 (9)	0.5490 (4)	0.0400 (7)	
C13	0.3191 (3)	0.40108 (10)	0.6711 (4)	0.0432 (7)	
H13	0.2707	0.4145	0.7344	0.052*	
C14	0.3452 (2)	0.36119 (9)	0.7016 (3)	0.0402 (7)	
C15	0.3034 (3)	0.34053 (11)	0.8295 (4)	0.0492 (8)	
H15	0.2612	0.3547	0.8986	0.059*	
C16	0.3231 (3)	0.30128 (11)	0.8533 (4)	0.0548 (9)	
H16	0.2945	0.2886	0.9379	0.066*	
C17	0.3869 (3)	0.27908 (11)	0.7506 (4)	0.0557 (9)	
H17	0.3987	0.2517	0.7666	0.067*	
C18	0.4313 (3)	0.29732 (10)	0.6286 (4)	0.0464 (8)	
H18	0.4737	0.2822	0.5626	0.056*	
C19	0.4146 (2)	0.33940 (9)	0.5989 (3)	0.0379 (7)	
C20	0.4605 (2)	0.35934 (9)	0.4753 (3)	0.0359 (7)	
C21	0.5279 (2)	0.33716 (8)	0.3628 (3)	0.0362 (7)	
C22	0.6302 (3)	0.32209 (9)	0.3981 (4)	0.0466 (8)	
H22	0.6588	0.3261	0.4938	0.056*	
C23	0.6905 (3)	0.30109 (10)	0.2926 (4)	0.0538 (9)	
H23	0.7593	0.2912	0.3180	0.065*	
C24	0.6502 (3)	0.29467 (10)	0.1514 (4)	0.0530 (9)	
H24	0.6904	0.2799	0.0817	0.064*	
C25	0.5496 (3)	0.31020 (11)	0.1135 (4)	0.0534 (9)	
H25	0.5224	0.3064	0.0169	0.064*	
C26	0.4887 (3)	0.33134 (10)	0.2176 (4)	0.0478 (8)	
H26	0.4208	0.3418	0.1905	0.057*	
S1	0.52265 (8)	0.49395 (3)	0.18065 (10)	0.0547 (3)	

### (II) 7-Phenylanthra[2,3-b]benzo[d]thiophene Atomic displacement parameters (Å^2^)

**Table:**

	*U*^11^	*U*^22^	*U*^33^	*U*^12^	*U*^13^	*U*^23^
C1	0.0346 (15)	0.0381 (15)	0.0371 (15)	0.0019 (12)	−0.0045 (13)	−0.0064 (13)
C2	0.0445 (17)	0.0402 (16)	0.0391 (17)	0.0081 (13)	−0.0050 (14)	−0.0058 (13)
C3	0.0466 (18)	0.0413 (17)	0.0388 (17)	0.0005 (14)	−0.0070 (14)	−0.0041 (13)
C4	0.0527 (19)	0.0462 (18)	0.0492 (19)	0.0015 (15)	−0.0137 (17)	−0.0067 (15)
C5	0.063 (2)	0.055 (2)	0.056 (2)	−0.0056 (18)	−0.0142 (18)	0.0020 (17)
C6	0.079 (3)	0.0383 (19)	0.083 (3)	−0.0056 (18)	−0.031 (2)	0.0072 (19)
C7	0.057 (2)	0.0421 (19)	0.089 (3)	0.0117 (16)	−0.016 (2)	−0.0154 (19)
C8	0.0470 (19)	0.0497 (19)	0.063 (2)	−0.0002 (15)	−0.0075 (16)	−0.0095 (17)
C9	0.0474 (18)	0.0382 (16)	0.0506 (19)	0.0015 (14)	−0.0136 (15)	−0.0088 (14)
C10	0.0424 (16)	0.0400 (16)	0.0425 (17)	0.0043 (13)	−0.0097 (14)	−0.0108 (14)
C11	0.0428 (17)	0.0422 (16)	0.0477 (19)	0.0079 (14)	−0.0018 (15)	−0.0117 (14)
C12	0.0371 (15)	0.0397 (16)	0.0431 (17)	0.0068 (12)	−0.0045 (13)	−0.0131 (13)
C13	0.0381 (16)	0.0488 (18)	0.0429 (17)	0.0036 (13)	0.0049 (14)	−0.0106 (14)
C14	0.0320 (15)	0.0461 (17)	0.0424 (17)	0.0001 (13)	−0.0032 (13)	−0.0085 (14)
C15	0.0404 (17)	0.064 (2)	0.0436 (18)	−0.0002 (15)	0.0069 (15)	−0.0034 (16)
C16	0.056 (2)	0.060 (2)	0.048 (2)	−0.0037 (17)	0.0068 (17)	0.0067 (17)
C17	0.063 (2)	0.0480 (19)	0.056 (2)	−0.0015 (16)	0.0022 (18)	0.0074 (17)
C18	0.0499 (19)	0.0428 (17)	0.0463 (18)	0.0039 (14)	0.0009 (15)	−0.0041 (14)
C19	0.0346 (15)	0.0414 (16)	0.0378 (16)	0.0013 (12)	−0.0052 (13)	−0.0063 (13)
C20	0.0337 (14)	0.0350 (14)	0.0390 (16)	0.0047 (12)	−0.0053 (12)	−0.0070 (12)
C21	0.0395 (16)	0.0313 (14)	0.0378 (16)	0.0015 (12)	−0.0001 (13)	−0.0026 (12)
C22	0.0503 (19)	0.0420 (17)	0.0473 (19)	0.0103 (14)	−0.0043 (15)	−0.0079 (14)
C23	0.0486 (19)	0.0469 (18)	0.066 (2)	0.0118 (15)	0.0052 (17)	−0.0024 (17)
C24	0.058 (2)	0.0461 (18)	0.055 (2)	0.0043 (16)	0.0195 (18)	−0.0078 (16)
C25	0.062 (2)	0.058 (2)	0.0407 (18)	−0.0048 (17)	0.0045 (16)	−0.0088 (16)
C26	0.0431 (17)	0.055 (2)	0.0450 (18)	0.0027 (15)	0.0013 (15)	−0.0070 (15)
S1	0.0709 (6)	0.0480 (5)	0.0453 (5)	0.0081 (4)	0.0074 (4)	0.0035 (4)

### (II) 7-Phenylanthra[2,3-b]benzo[d]thiophene Geometric parameters (Å, º)

**Table:**

C1—C20	1.414 (4)	C13—H13	0.9300
C1—C2	1.414 (4)	C14—C15	1.423 (5)
C1—C12	1.444 (4)	C14—C19	1.440 (4)
C2—C3	1.362 (4)	C15—C16	1.338 (5)
C2—H2	0.9300	C15—H15	0.9300
C3—C10	1.431 (4)	C16—C17	1.408 (5)
C3—S1	1.734 (3)	C16—H16	0.9300
C4—C5	1.380 (5)	C17—C18	1.356 (5)
C4—C9	1.385 (5)	C17—H17	0.9300
C4—S1	1.764 (4)	C18—C19	1.433 (4)
C5—C6	1.366 (6)	C18—H18	0.9300
C5—H5	0.9300	C19—C20	1.400 (4)
C6—C7	1.384 (6)	C20—C21	1.489 (4)
C6—H6	0.9300	C21—C22	1.382 (4)
C7—C8	1.388 (5)	C21—C26	1.392 (4)
C7—H7	0.9300	C22—C23	1.381 (5)
C8—C9	1.393 (5)	C22—H22	0.9300
C8—H8	0.9300	C23—C24	1.366 (5)
C9—C10	1.466 (4)	C23—H23	0.9300
C10—C11	1.350 (5)	C24—C25	1.374 (5)
C11—C12	1.424 (4)	C24—H24	0.9300
C11—H11	0.9300	C25—C26	1.380 (5)
C12—C13	1.386 (5)	C25—H25	0.9300
C13—C14	1.386 (4)	C26—H26	0.9300
			
C20—C1—C2	122.0 (3)	C13—C14—C19	119.2 (3)
C20—C1—C12	119.5 (3)	C15—C14—C19	118.6 (3)
C2—C1—C12	118.5 (3)	C16—C15—C14	121.9 (3)
C3—C2—C1	119.9 (3)	C16—C15—H15	119.0
C3—C2—H2	120.1	C14—C15—H15	119.0
C1—C2—H2	120.1	C15—C16—C17	120.2 (3)
C2—C3—C10	121.9 (3)	C15—C16—H16	119.9
C2—C3—S1	125.2 (3)	C17—C16—H16	119.9
C10—C3—S1	112.9 (2)	C18—C17—C16	120.6 (3)
C5—C4—C9	121.9 (3)	C18—C17—H17	119.7
C5—C4—S1	125.7 (3)	C16—C17—H17	119.7
C9—C4—S1	112.3 (3)	C17—C18—C19	121.6 (3)
C6—C5—C4	118.3 (4)	C17—C18—H18	119.2
C6—C5—H5	120.9	C19—C18—H18	119.2
C4—C5—H5	120.9	C20—C19—C18	123.1 (3)
C5—C6—C7	121.4 (3)	C20—C19—C14	119.9 (3)
C5—C6—H6	119.3	C18—C19—C14	117.0 (3)
C7—C6—H6	119.3	C19—C20—C1	120.0 (3)
C6—C7—C8	120.3 (3)	C19—C20—C21	121.1 (3)
C6—C7—H7	119.9	C1—C20—C21	118.8 (3)
C8—C7—H7	119.9	C22—C21—C26	118.2 (3)
C7—C8—C9	118.9 (4)	C22—C21—C20	121.7 (3)
C7—C8—H8	120.6	C26—C21—C20	120.1 (3)
C9—C8—H8	120.6	C23—C22—C21	120.6 (3)
C8—C9—C4	119.3 (3)	C23—C22—H22	119.7
C8—C9—C10	127.7 (3)	C21—C22—H22	119.7
C4—C9—C10	113.0 (3)	C22—C23—C24	120.8 (3)
C11—C10—C3	119.5 (3)	C22—C23—H23	119.6
C11—C10—C9	130.2 (3)	C24—C23—H23	119.6
C3—C10—C9	110.3 (3)	C25—C24—C23	119.3 (3)
C10—C11—C12	120.8 (3)	C25—C24—H24	120.3
C10—C11—H11	119.6	C23—C24—H24	120.3
C12—C11—H11	119.6	C24—C25—C26	120.5 (3)
C13—C12—C11	121.7 (3)	C24—C25—H25	119.8
C13—C12—C1	119.1 (3)	C26—C25—H25	119.8
C11—C12—C1	119.2 (3)	C25—C26—C21	120.5 (3)
C12—C13—C14	122.0 (3)	C25—C26—H26	119.7
C12—C13—H13	119.0	C21—C26—H26	119.7
C14—C13—H13	119.0	C3—S1—C4	91.43 (16)
C13—C14—C15	122.2 (3)		
			
C20—C1—C2—C3	177.9 (3)	C13—C14—C15—C16	175.5 (3)
C12—C1—C2—C3	−3.1 (4)	C19—C14—C15—C16	−2.7 (5)
C1—C2—C3—C10	−1.6 (5)	C14—C15—C16—C17	0.1 (5)
C1—C2—C3—S1	178.5 (2)	C15—C16—C17—C18	1.6 (6)
C9—C4—C5—C6	−1.4 (5)	C16—C17—C18—C19	−0.5 (5)
S1—C4—C5—C6	179.9 (3)	C17—C18—C19—C20	179.0 (3)
C4—C5—C6—C7	0.8 (6)	C17—C18—C19—C14	−2.1 (5)
C5—C6—C7—C8	0.1 (6)	C13—C14—C19—C20	4.3 (4)
C6—C7—C8—C9	−0.5 (5)	C15—C14—C19—C20	−177.5 (3)
C7—C8—C9—C4	−0.1 (5)	C13—C14—C19—C18	−174.7 (3)
C7—C8—C9—C10	−179.9 (3)	C15—C14—C19—C18	3.6 (4)
C5—C4—C9—C8	1.1 (5)	C18—C19—C20—C1	178.8 (3)
S1—C4—C9—C8	180.0 (2)	C14—C19—C20—C1	−0.1 (4)
C5—C4—C9—C10	−179.2 (3)	C18—C19—C20—C21	2.0 (4)
S1—C4—C9—C10	−0.2 (3)	C14—C19—C20—C21	−176.9 (3)
C2—C3—C10—C11	3.8 (5)	C2—C1—C20—C19	174.5 (3)
S1—C3—C10—C11	−176.4 (2)	C12—C1—C20—C19	−4.5 (4)
C2—C3—C10—C9	−177.4 (3)	C2—C1—C20—C21	−8.6 (4)
S1—C3—C10—C9	2.5 (3)	C12—C1—C20—C21	172.4 (3)
C8—C9—C10—C11	−3.0 (6)	C19—C20—C21—C22	−69.3 (4)
C4—C9—C10—C11	177.3 (3)	C1—C20—C21—C22	113.8 (3)
C8—C9—C10—C3	178.4 (3)	C19—C20—C21—C26	111.1 (3)
C4—C9—C10—C3	−1.4 (4)	C1—C20—C21—C26	−65.7 (4)
C3—C10—C11—C12	−1.0 (5)	C26—C21—C22—C23	−1.4 (5)
C9—C10—C11—C12	−179.6 (3)	C20—C21—C22—C23	179.0 (3)
C10—C11—C12—C13	176.1 (3)	C21—C22—C23—C24	−0.2 (5)
C10—C11—C12—C1	−3.7 (4)	C22—C23—C24—C25	1.6 (5)
C20—C1—C12—C13	4.9 (4)	C23—C24—C25—C26	−1.4 (5)
C2—C1—C12—C13	−174.1 (3)	C24—C25—C26—C21	−0.2 (5)
C20—C1—C12—C11	−175.3 (3)	C22—C21—C26—C25	1.6 (5)
C2—C1—C12—C11	5.7 (4)	C20—C21—C26—C25	−178.8 (3)
C11—C12—C13—C14	179.5 (3)	C2—C3—S1—C4	177.6 (3)
C1—C12—C13—C14	−0.7 (5)	C10—C3—S1—C4	−2.2 (2)
C12—C13—C14—C15	177.9 (3)	C5—C4—S1—C3	−179.7 (3)
C12—C13—C14—C19	−3.9 (5)	C9—C4—S1—C3	1.4 (3)

### (II) 7-Phenylanthra[2,3-b]benzo[d]thiophene Hydrogen-bond geometry (Å, º)

Cg2 and Cg3 are the centroids of rings (C1–C3/C10–C12) and (C1/C12–C14/C19/C20), respectively.

**Table:**

*D*—H···*A*	*D*—H	H···*A*	*D*···*A*	*D*—H···*A*
C13—H13···*Cg*2^i^	0.93	2.97	3.885 (4)	168
C15—H15···*Cg*3^i^	0.93	2.57	3.479 (4)	166

Symmetry code: (i) *x*, −*y*−1/2, *z*−1/2.

## References

[bb1] Bedworth, P. V., Cai, Y., Jen, A. & Marder, S. R. (1996). *J. Org. Chem.* **61**, 2242–2246.

[bb2] Bonini, C., Chiummiento, L., Bonis, M. D., Funicello, M., Lupattelli, P., Suanno, G., Berti, F. & Campaner, P. (2005). *Tetrahedron*, **61**, 6580–6589.

[bb3] Brault, L., Migianu, E., Néguesque, A., Battaglia, E., Bagrel, D. & Kirsch, G. (2005). *Eur. J. Med. Chem.* **40**, 757–763. 10.1016/j.ejmech.2005.02.010 16122578

[bb4] Bruker (2008). *APEX2*, *SAINT* and *SADABS*. Bruker AXS Inc., Madison, Wisconsin, USA.

[bb5] Dhayalan, V., Clement, J. A., Jagan, R. & Mohanakrishnan, A. K. (2009). *Eur. J. Org. Chem.* pp. 531–546.

[bb6] Du, C., Guo, Y., Liu, Y., Qiu, W., Zhang, H., Gao, X., Liu, Y., Qi, T., Lu, K. & Yu, G. (2008). *Chem. Mater.* **20**, 4188–4190.

[bb7] Farrugia, L. J. (2012). *J. Appl. Cryst.* **45**, 849–854.

[bb8] Groom, C. R., Bruno, I. J., Lightfoot, M. P. & Ward, S. C. (2016). *Acta Cryst.* B**72**, 171–179. 10.1107/S2052520616003954 PMC4822653 27048719

[bb9] Hänsel, H., Zettl, H., Krausch, G., Kisselev, R., Thelakkat, M. & Schmidt, H. W. (2003). *Adv. Mater.* **15**, 2056–2060.

[bb10] Jordan, V. C. (2003). *J. Med. Chem.* **46**, 1081–1111. 10.1021/jm020450x 12646017

[bb11] Justin Thomas, K. R., Hsu, Y. C., Lin, J. T., Lee, K. M., Ho, K. C., Lai, C. H., Cheng, Y. M. & Chou, P. T. (2008). *Chem. Mater.* **20**, 1830–1840.

[bb12] Li, G., Zhou, S., Su, G., Liu, Y. & Wang, P. G. (2007). *J. Org. Chem.* **72**, 9830–9833. 10.1021/jo7017334 17994762

[bb13] Macrae, C. F., Bruno, I. J., Chisholm, J. A., Edgington, P. R., McCabe, P., Pidcock, E., Rodriguez-Monge, L., Taylor, R., van de Streek, J. & Wood, P. A. (2008). *J. Appl. Cryst.* **41**, 466–470.

[bb14] Mazzeo, M., Vitale, V., Della Sala, F., Pisignano, D., Anni, M., Barbarella, G., Favaretto, L., Zanelli, A., Cingolani, R. & Gigli, G. (2003). *Adv. Mater.* **15**, 2060–2063.

[bb15] Rafiq, S. M., Sivasakthikumaran, R. & Mohanakrishnan, A. K. (2014). *Org. Lett.* **16**, 2720–2723. 10.1021/ol501006t 24773051

[bb16] Raposo, M. M. M., Fonseca, A. M. C., Castro, M. C. R., Belsley, M., Cardoso, M. F. S., Carvalho, L. M. & Coelho, P. J. (2011). *Dyes Pigments*, **91**, 62–73.

[bb17] Russell, R. K. & Press, J. B. (1996). *Comprehensive Heterocyclic Chemistry II*, Vol. 2, edited by A. R. Katritzky, C. W. Rees & E. F. V. Scriven. pp. 679–729. Oxford: Pergamon Press.

[bb18] Sheldrick, G. M. (2008). *Acta Cryst.* A**64**, 112–122. 10.1107/S0108767307043930 18156677

[bb19] Silambarasan, V., Srinivasan, T., Sivasakthikumaran, R., Mohanakrishnan, A. K. & Velmurugan, D. (2012). *Acta Cryst.* E**68**, o3408–o3409. 10.1107/S1600536812047137 PMC3588996 23476232

[bb20] Silambarasan, V., Srinivasan, T., Sivasakthikumaran, R., Mohana­krishnan, A. K. & Velmurugan, D. (2013). *Acta Cryst.* E**69**, o36. 10.1107/S1600536812049471 PMC3588245 23476423

[bb21] Sivasakthikumaran, R., Nandakumar, M. & Mohanakrishnan, A. K. (2012). *J. Org. Chem.* **77**, 9053–9071. 10.1021/jo301410w 22974723

[bb22] Spek, A. L. (2009). *Acta Cryst.* D**65**, 148–155. 10.1107/S090744490804362X PMC2631630 19171970

[bb23] Zhan, X., Tan, Z.-A., Domercq, B., An, Z., Zhang, X., Barlow, S., Li, Y.-F., Zhu, D.-B., Kippelen, B. & Marder, S. R. (2007). *J. Am. Chem. Soc.* **129**, 7246–7247. 10.1021/ja071760d 17508752

[bb24] Zuse, A., Schmidt, P., Baasner, S., Böhm, K. J., Müller, K., Gerlach, M., Günther, E. G., Unger, E. & Prinz, H. (2006). *J. Med. Chem.* **49**, 7816–7825. 10.1021/jm0605031 17181164

[bb25] Zuse, A., Schmidt, P., Baasner, S., Böhm, K. J., Müller, K., Gerlach, M., Günther, E. G., Unger, E. & Prinz, H. (2007). *J. Med. Chem.* **50**, 6059–6066. 10.1021/jm0708984 17973361

